# Surgical Resection of a Leiomyosarcoma of the Inferior Vena Cava Mimicking Hepatic Tumor

**DOI:** 10.1155/2013/235698

**Published:** 2013-02-20

**Authors:** Junji Ueda, Hiroshi Yoshida, Yasuhiro Mamada, Nobuhiko Taniai, Masato Yoshioka, Youichi Kawano, Yoshiaki Mizuguchi, Tetsuya Shimizu, Hideyuki Takata, Eiji Uchida

**Affiliations:** ^1^Department of Gastrointestinal Hepatobiliary-Pancreatic Surgery, Nippon Medical School, Tokyo 113-8603, Japan; ^2^Department of Surgery for Organ Function and Biological Regulation, Graduate School of Medicine, Nippon Medical School, Tokyo, Japan

## Abstract

*Introduction*. Leiomyosarcomas of vascular origin are particularly rare tumors occurring mainly in the inferior vena cava (IVC). They are malignant, slow-growing tumors with a poor prognosis. This paper reports on a rare case of surgical resection of an IVC leiomyosarcoma mimicking a hepatic tumor. *Case Presentation*. A 65-year-old Japanese male was admitted for evaluation of an abdominal tumor. Enhanced computed tomography of the abdomen revealed a slightly enhanced heterogeneous tumor, 18 mm in diameter, between the Spiegel lobe of the liver and the IVC in early-phase images, with no enhancement or washout in late-phase images. We diagnosed this tumor as either a hepatic tumor in the Spiegel lobe or a retroperitoneal tumor such as leiomyosarcoma or liposarcoma and performed a laparotomy. On the basis of surgical findings, we extirpated the tumor by performing a wedge resection of the wall of the IVC and suturing the primary IVC wall. Pathological findings led to a further diagnosis of the tumor as a leiomyosarcoma originating in the IVC. Thirty-seven months after the operation, multiple liver and lung metastases were detected, and the patient died from multiple organic failures. *Conclusion*. We experienced a rare case of a leiomyosarcoma of IVC mimicking hepatic tumor.

## 1. Introduction

Leiomyosarcomas are malignant tumors of smooth muscle cells that can originate in any location but occur most often in the uterus, retroperitoneum, or intraabdominal region [1]. Leiomyosarcomas of vascular origin are particularly rare tumors occurring mainly in the inferior vena cava (IVC) [2, 3]. The International Registry of Inferior Vena Cava Leiomyosarcomas revealed that most tumors occur in the lower (44.2%) or middle (50.8%) region of the IVC, while only a small number of tumors occur in the upper third or suprahepatic region (4.2%) [4]. Leiomyosarcoma of IVC was first described by Dzsinich et al. in 1992 [5]. There are no definitive symptoms, but typical symptoms include dyspnea and a general feeling of being unwell accompanied by weight loss and abdominal and/or back pain [6]. A correct diagnosis is difficult, and, in up to one-third of cases, it is not made until after a postmortem examination [3]. Diagnosis is made using modern imaging techniques such as ultrasound, echocardiography, computed tomography (CT), and magnetic resonance imaging (MRI) [2]. In spite of the advancement of modern imaging techniques, it is difficult to diagnose a leiomyosarcoma preoperatively, and in some cases it is also difficult to distinguish it from a hepatic tumor [4]. This paper reports on a rare case of surgical resection of an IVC leiomyosarcoma mimicking a hepatic tumor.

## 2. Case Presentation

A 65-year-old Japanese male was admitted for evaluation of an abdominal tumor. The tumor was detected by chance during a chest CT performed for a lung examination. The patient had no past history of any abdominal surgery, and his vital signs were stable. There is nothing particularly significant in the findings of the laboratory examination. Tumor markers are negative.

**Figure 1:**
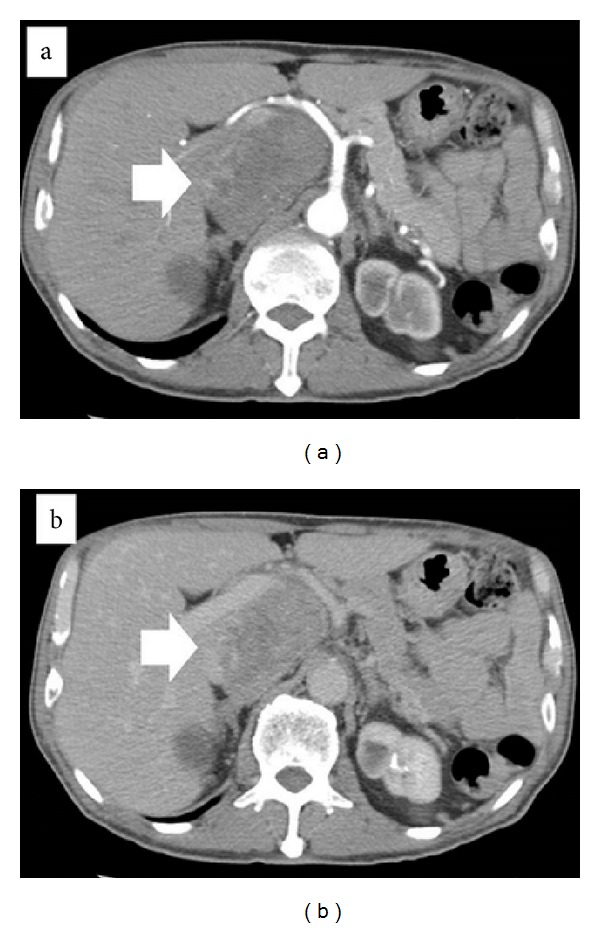
Enhanced computed tomography (CT) of the abdomen revealed a slightly enhanced heterogeneous tumor, 18 mm in diameter, between the Spiegel lobe of the liver and the IVC in early-phase images ((a) arrow), with no enhancement and washout in late-phase images ((b) arrow).

**Figure 2:**
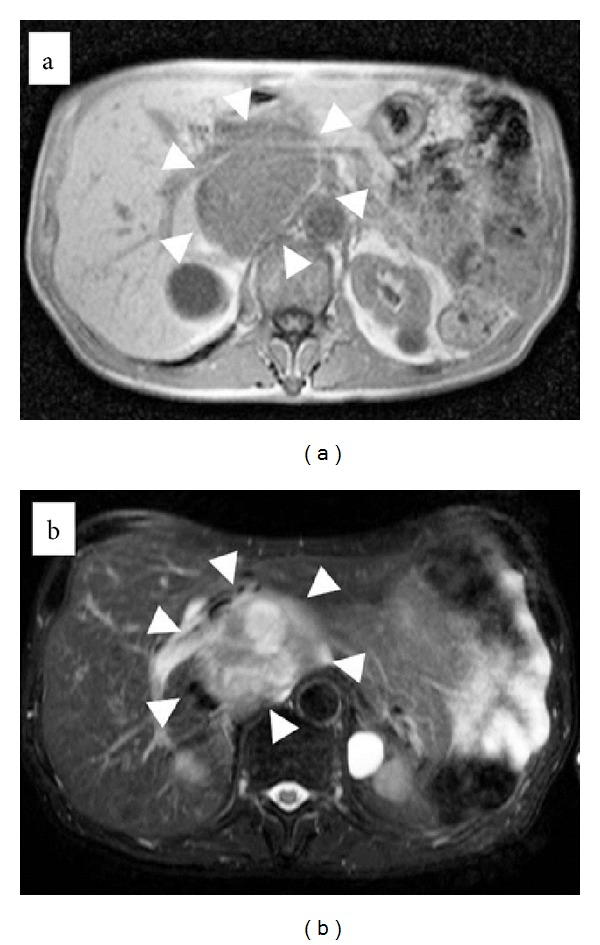
Magnetic resonance imaging (MRI) of the abdomen. Magnetic resonance imaging (MRI) of this tumor revealed a contrasting low intensity on the T1-weighted image ((a) arrow head) and high intensity on the T2-weighted image ((b) arrow head).

Enhanced computed tomography of the abdomen revealed a slightly enhanced heterogeneous tumor, 18 mm in diameter, between the Spiegel lobe of the liver and the IVC in early-phase images (Figure 1(a)), with no enhancement or washout in late-phase images (Figure 1(b)). Magnetic resonance imaging of this tumor revealed a contrasting low intensity on the T1-weighted image (Figure 2(a)) and high intensity on the T2-weighted image (Figure 2(b)). Upper-gastrointestinal endoscopy and colonoscopy revealed no evidence of a malignant tumor in the gastrointestinal tract. We diagnosed this tumor as either a hepatic tumor in the Spiegel lobe or a retroperitoneal tumor such as leiomyosarcoma or liposarcoma.

**Figure 3:**
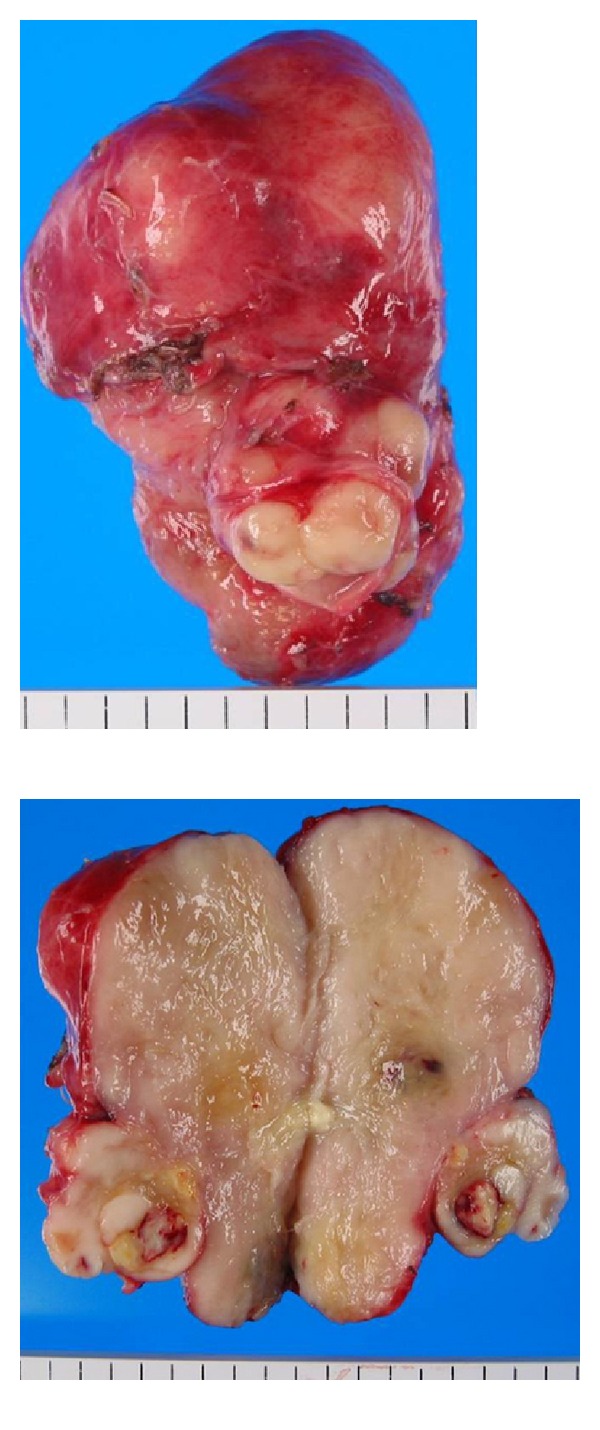
The resected specimen was solid with a smooth surface.

**Figure 4:**
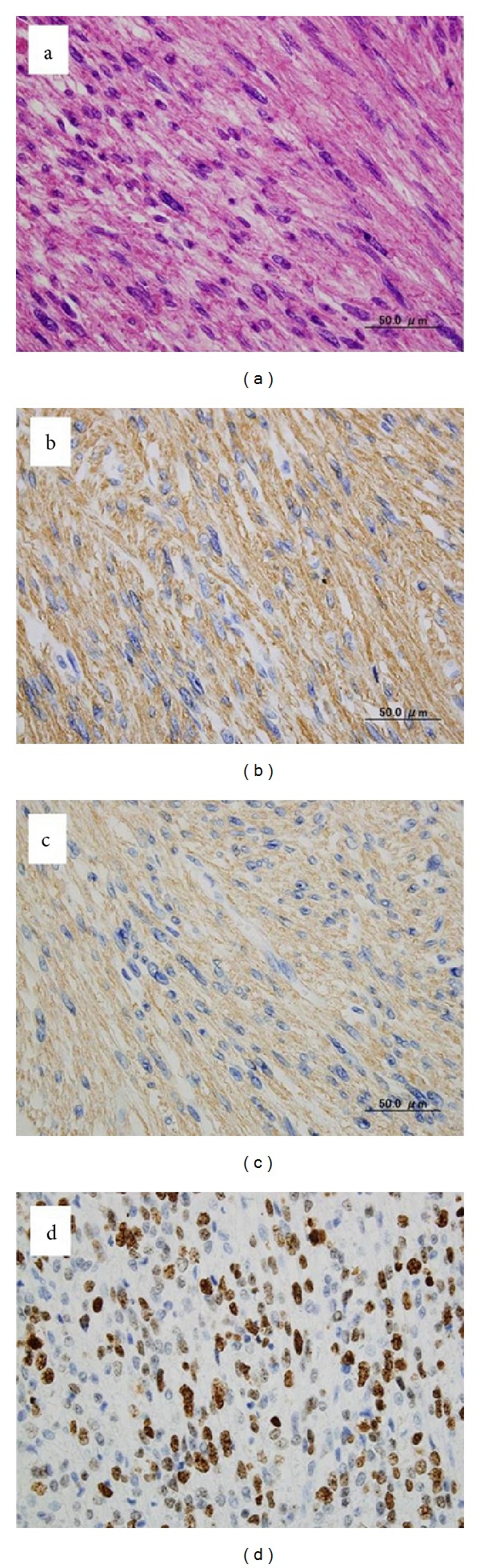
Histopathological findings. Microscopic examination revealed that the tumor consisted of uniform and spindle cells and had a fascicular growth pattern (Hematoxylin & Eosin: ×600) (a). Immunohistochemical staining revealed that *α*-SMA (×600) (b) and HHF35 (×600) (c) were expressed in this tumor; MIB-1 index was about 60% (Ki67: ×600) (d).

**Figure 5:**
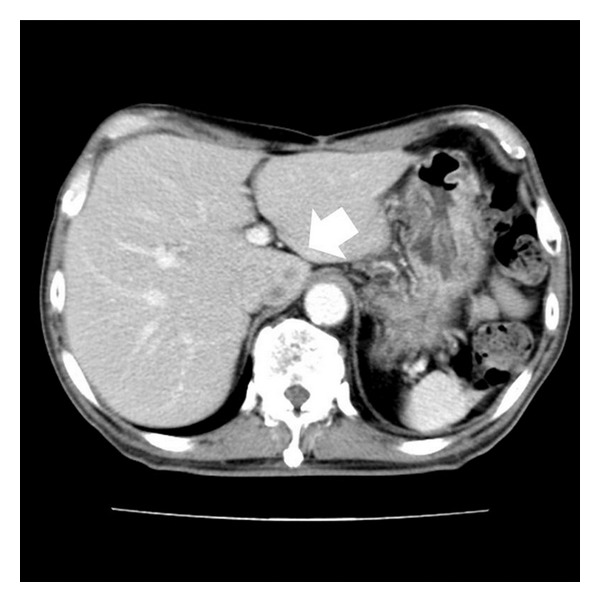
Enhanced computed tomography (CT) of the abdomen. The tumor was detected in the Spiegel of the liver on enhanced CT of the abdomen (arrow).

**Figure 6:**
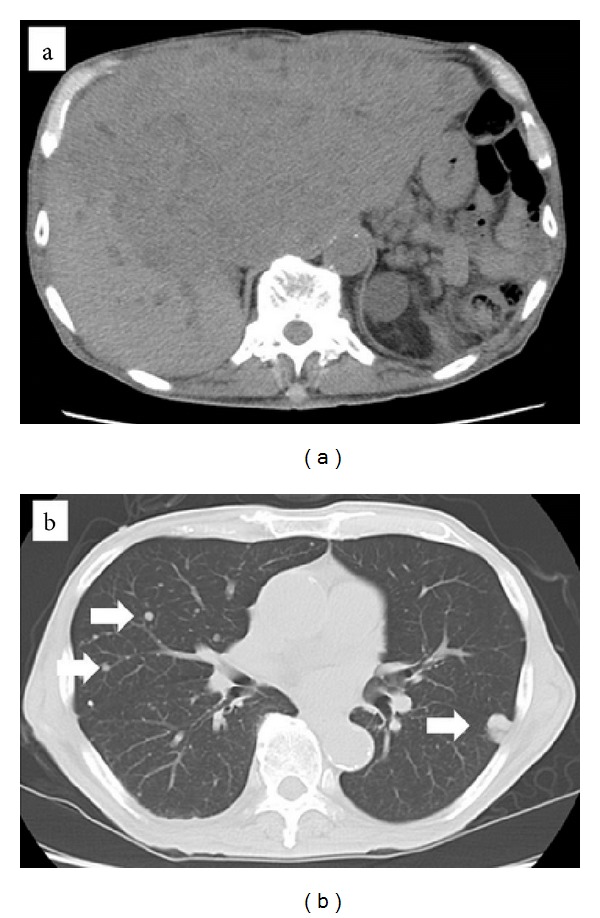
Computed tomography (CT) of the abdomen and chest. Multiple liver metastases (a) and lung metastases ((b) arrows) were detected on CT.

A laparotomy was performed, and the surface of the tumor was found to be smooth and slightly adhesive. To expose the affected IVC lesion, we need to mobilize some parts of the right lobe of the liver. This tumor was not located in the liver but originated in the IVC. We extirpated the tumor by performing a wedged resection of the wall of the IVC and sutured primary IVC wall. We controlled the bleeding from the IVC using the hemostatic forceps. The resected specimen was solid with a smooth surface (Figure 3). Microscopic examination revealed that the tumor consisted of uniform and spindle cells and had a fascicular growth pattern. This pathological feature was compatible with a mesenchymal tumor (Figure 4(a)). Immunohistochemical staining revealed that *α*-SMA and HHF35 were expressed in this tumor (Figures 4(b) and 4(c)), and c-kit and CD34 were negative. The MIB-1 index was about 60% (Figure 4(d)). The tumor was diagnosed as a leiomyosarcoma originating in the IVC. The patient's recovery was uneventful, and he was discharged on postoperative day 10. After 12 months, the tumor was detected in the Spiegel of the liver on enhanced CT of the abdomen (Figure 5). We diagnosed it as a recurrence of the leiomyosarcoma. We suggested to the patient and his family that he should undergo additional treatments such as chemotherapy, radiation, or surgical resection, but they declined this. Thirty-seven months after the operation, multiple liver and lung metastases were detected on CT (Figures 6(a) and 6(b)), and the patient died of multiple organic failures.

## 3. Discussion

Leiomyosarcomas originating in the IVC are rare, malignant, slow-growing tumors with a poor prognosis [6]. Tumor metastases of this slow-growing malignant tumor to other organs are relatively infrequent, but can be detected in the liver, lungs, lymph nodes, or bones [7, 8]. In this case, there were no symptoms, and the tumor was detected by chance in a chest CT. Present day imaging techniques such as abdominal ultrasonography, echocardiography, CT, and MRI are commonly employed to make a rapid and precise diagnosis of a suspected leiomyosarcoma [6]. In this case we could not preoperatively diagnose the tumor as leiomyosarcoma of the IVC and also could not rule out a potential liver tumor. A final conclusive diagnosis is of course made by means of histopathological and immunohistochemical methods. The pathognomonic findings of leiomyosarcoma are spindle-shaped tumor cells with positive markers for smooth muscle cells, vimentin, muscle actin, alpha-smooth muscle actin, and desmin [9]. 

The recommended therapy for treating leiomyosarcoma is aggressive surgical removal of the tumor by means of modern vascular surgery, in combination with chemotherapy and/or radiotherapy [2, 8, 10]. In surgery, a complete resection of the tumor was possible, and the IVC was repaired primarily via surgical means without too high a risk of postoperative edema. For leiomyosarcomas, there is a perioperative mortality of 4%, and 42% of the patients died of the disease itself [11]. To reduce the tumor size and increase the resection rate, a preoperative neoadjuvant therapy can be implemented. If, however, a complete tumor resection is not possible, tumor reduction followed by radiation therapy provides a good palliative treatment option [12]. 

Postoperative therapy and the management of recurrence are difficult because of the absence of evidence for their effectiveness in patients. Radiation has been used in both the neoadjuvant and adjuvant settings, and some authors believe that it may help with the local control of disease [13]. For localized, resectable, soft-tissue sarcomas, adjuvant chemotherapy with doxorubicin or a combination of doxorubicin and ifosfamide has been shown to prolong the time before recurrence and the overall rate of survival [14]. This therapy may thus also be effective in the treatment of IVC leiomyosarcoma. A case has been previously reported in which an IVC leiomyosarcoma with liver metastasis was positive for steroid receptors, including the estrogen receptor (ER) and progesterone receptor (PR) [4]. The patient received combined multimodal therapy, including resection of the tumor, intrahepatic arterial chemotherapy, and hormonal manipulation. Thus hormonal manipulation using medroxyprogesterone and/or tamoxifen should be tried in ER/PR-positive cases. Although there is no established treatment for patients with IVC leiomyosarcoma, we hope that thorough investigation of this condition will help establish a standard treatment yielding satisfactory results [4]. In a series of case studies of 14 patients with a leiomyosarcoma of the IVC who underwent generous resection followed by radiation, it was established that radiotherapy reduced local recurrence and increased the median survival time. In addition, a combination of chemotherapy and radiotherapy has been reported as being superior to radiotherapy alone with respect to an increase in the survival rate [13]. The long-term outcome of surgery for leiomyosarcoma of the IVC has been disappointing. Some reports with sufficient case numbers and followup revealed that a 5-year survival rate was achieved 33%–53% after radical resection followed by a curative approach using adjuvant therapy [2, 8, 13]. We should diagnose this tumor in early stage, and aggressive surgical management using modern vascular surgical and oncology techniques.

## 4. Conclusion 

We encountered a rare case of leiomyosarcoma of the IVC mimicking a liver tumor. We must always consider leiomyosarcoma when treating a tumor between the liver and the IVC and be prepared to perform a complete surgical resection in the event of leiomyosarcoma and follow up carefully. We must also consider adjuvant therapy for a recurrent leiomyosarcoma.
